# Copy number variations in urine cell free DNA as biomarkers in advanced prostate cancer

**DOI:** 10.18632/oncotarget.9027

**Published:** 2016-04-26

**Authors:** Yun Xia, Chiang-Ching Huang, Rachel Dittmar, Meijun Du, Yuan Wang, Hongyan Liu, Niraj Shenoy, Liang Wang, Manish Kohli

**Affiliations:** ^1^ Department of General Surgery, Tongji Hospital of Tongji Medical College, Huazhong University of Science and Technology, Wuhan, China; ^2^ Department of Pathology and MCW Cancer Center, Medical College of Wisconsin, Milwaukee, WI, USA; ^3^ Joseph J. Zilber School of Public Health, University of Wisconsin, Milwaukee, WI, USA; ^4^ Division of Medical Oncology, Department of Oncology, Mayo Clinic, Rochester, MN, USA

**Keywords:** prostate cancer, liquid biopsy, urine, cell free DNA, next generation sequencing

## Abstract

Genetic profiling of urine cell free DNA (cfDNA) has not been evaluated in advanced prostate cancer. We performed whole genome sequencing of urine cfDNAs to identify tumor-associated copy number variations in urine before and after initiating androgen deprivation therapy in HSPC stage and docetaxel chemotherapy in CRPC stage. A log2 ratio-based copy number analysis detected common genomic abnormalities in prostate cancer including *AR* amplification in 5/10 CRPC patients. Other abnormalities identified included *TMPRSS2-ERG* fusion, *PTEN* gene deletion, *NOTCH1* locus amplification along with genomic amplifications at 8q24.3, 9q34.3, 11p15.5 and 14q11.2, and deletions at 4q35.2, 5q31.3, 7q36.3, 12q24.33, and 16p11.2. By comparing copy number between pre- and post-treatment, we found significant copy number changes in 34 genomic loci. To estimate the somatic tumor DNA fraction in urine cfDNAs, we developed a Urine Genomic Abnormality (UGA) score algorithm that summed the top ten most significant segments with copy number changes. The UGA scores correlated with tumor burden and the change in UGA score after stage-specific therapies reflected disease progression status and overall survival. The study demonstrates the potential clinical utility of urine cfDNAs in predicting treatment response and monitoring disease progression.

## INTRODUCTION

Prostate cancer is the most common non-skin cancer among US men with 220,800 new cases estimated in 2015 and more than 27,500 projected deaths [1]. Recently it has been shown that addition of docetaxel chemotherapy prolongs survival in this stage [2]. Despite this advance, emergence of castration-resistant prostate cancer (CRPC) is inevitable and is treated with systemic chemotherapy with docetaxel [3–7] and several novel systemic anti-cancer therapies [8–17]. Unfortunately, predictive biomarkers for response, efficacy or toxicity to traditional or novel treatments in advanced prostate cancer therapeutics are lacking and the practice continues to be based on best clinical estimates. Molecular classifiers of disease outcome or therapeutic benefit and toxicity are needed for individualizing therapeutic choices.

Body fluid-based biomarkers are appealing in advanced prostate cancer because they are less invasive and easily accessible. Cell free DNA (cfDNA)-based somatic aberrations in plasma of cancer patients have been extensively reported [15, 18, 19]. In advanced prostate cancer patients tumor-derived plasma cfDNA is detected in hormone sensitive and castrate resistant stages [20]. It remains unclear if genomic profiling to detect tumor cfDNA aberrations in urine is feasible and clinically relevant for developing as predictive biomarkers. To determine somatic genomic changes we performed whole-genome sequencing and analyzed copy number variations in matched urine specimens of advanced prostate cancer patients previously sequenced for plasma cfDNA [20]. We first evaluated the urine genome abnormality (UGA) algorithm based on genome-wide copy number variation (CNVs) to determine association with treatment response and clinical outcomes in patients receiving standard advanced prostate cancer treatments. We then compared urine cfDNA-based CNVs with previously reported plasma cfDNA CNVs [20]. Our data show that urine cfDNAs may generate comparable results to plasma cfDNA in CNVs analysis and may have clinical application in predicting treatment response and clinical outcomes.

## RESULTS

### Patients' clinical characteristics

Matched urine specimens for patients with previous cfDNA sequencing of plasma specimens were available for 9 of 10 hormone sensitive prostate cancer (HSPC) patients and all ten patients with castrate resistant prostate cancer (CRPC) disease. For this study we selected these samples for urine cfDNA purification. Patient characteristics for these two advanced cancer cohorts are presented in Table 1. Each subject had two serial urine specimens collected before and after initiating stage-specific treatments. All patients in the HSPC sub-cohort underwent continuous ADT and patients with CRPC received docetaxel chemotherapy in addition to ADT as standard stage-specific treatments. The mean time between two sample collections in the HSPC was 128 days; the mean time between two sample collections in the CRPC was 112.4 days. The median follow-up was 64.00 months (40.93-69.13months) and 20.97 months (range 6.77-72.83 months) for HSPC and CRPC cohorts, respectively.

**Table 1:** Clinical characteristics of 19 advanced prostate cancer patients

Patient ID	Advanced Prostate Cancer Sub Cohort	Advanced PCA Sub Cohort Standard of care treatment	Age at Initial Diagnosis (years)	TNM staging at initial diagnosis	Initial Gleason Score (GS)	Initial PCA treatment	Time from initial PCA diagnosis to development of HSPC (months)	Time from initial PCA diagnosis to development of CRPC (months)	Time from initiating ADT for HSPC stage to development of CRPC (months)	Metastatic volume status before initiating treatments*	PSA (ng/ml) at time of 1st sample collection in advanced sub cohort stage	PSA (ng/ml) at time of 2nd sample collection in advanced stage	Time (days) between two sample collections	Follow-up time (months)	Vital status Alive=0 Dead=1
1001	CRPC	Chemo	62	T4N1M1	9	ADT	0	7	7	High Volume	8.2	0.42	147	37.07	0
1002	CRPC	Chemo	66	T2cNxM1	NA	ADT	0	27	27	Low Volume	9.3	1.6	89	17.47	1
1003	CRPC	Chemo	54	T3aN0M0	7	Radical Prostatectomy	52	59	7	High Volume	107	162	84	6.77	1
1004	CRPC	Chemo	69	T3aNxM0	8	Radical Prostatectomy	18	60	42	Low Volume	3.4	4.6	92	59.07	0
1005	CRPC	Chemo	69	T3bN2M1	9	ADT	0	8	8	High Volume	0.48	0.1	146	9.53	1
1010	CRPC	Chemo	72	T3bN1M0	9	Radical Prostatectomy+ADT+Chemotherapy	0	50	50	High Volume	5	NA	144	21.83	1
1014	CRPC	Chemo	61	T2bN1M1	7	ADT	0	4	4	High Volume	126	56.8	99	20.1	1
1017	CRPC	Chemo	63	T2aN0M0	5	Radical Prostatectomy	12	206	194	Low Volume	22	104	139	56.7	0
1043	CRPC	Chemo	73	T2aNxM1	7	ADT	0	37	37	High Volume	15.5	8	80	33.1	0
1060	CRPC	Chemo	78	TxNxM1	NA	ADT	0	9	9	High Volume	3.7	1.4	104	18.93	1
1015	HSPC	ADT	67	T2cNxM0	7	Radical Prostatectomy	30	NR	NR	Low Volume	1	0.9	98	54.23	0
1028	HSPC	ADT	49	T3bN0M0	9	Radical Prostatectomy+ADT	0	96	96	Low Volume	0.33	0.12	154	47.67	0
1040	HSPC	ADT	53	T2NxM0	9	Laparoscopic Prostatectomy	10	NR	NR	Low Volume	2.5	<0.10	168	40.93	0
1050	HSPC	ADT	64	T3bN1M1	9	Radiation+ADT	0	48	48	Low Volume	4.2	<0.10	136	55.13	0
1059	HSPC	ADT	62	T3bN1M0	9	Radical Prostatectomy+ADT	0	NR	NR	Low Volume	2.9	<0.10	116	54.23	0
1080	HSPC	ADT	65	T3bN1M0	8	Radiation+ADT	0	NR	NR	Low Volume	16	0.77	172	54.86	0
1084	HSPC	ADT	57	T3bN0M0	9	Radical Prostatectomy	2	8	6	High Volume	2.2	0.24	78	53.07	0
1098	HSPC	ADT	78	T2aNxM0	6	External Beam Radiation Therapy	60	NR	NR	Low Volume	5.7	0.54	131	51.57	0
1104	HSPC	ADT	67	T2cN1M1	8	ADT+Chemotherapy	0	12	12	Low Volume	37	<0.10	99	52	0

* High volume metastatic disease is defined as the presence of non-nodal visceral metastasis or 4 or more skeletal lesions with at least one outside the axial skeleton

Abbrevation: NR: Not Reached

ADT: Androgen Deprivation Therapy

PCA: Prostate Cancer

### Urine cfDNA yield and quality

To assess cfDNA yield, we tested three different kits using one single urine sample. We found that average cfDNA yields from 15 ml urine were 5.63ng, 6.46ng and 13.27ng for Zymo, Norgen and Analytik, respectively (Supplementary Figure 1). The Analytik kit generated approximately two fold more cfDNA than the two other kits. Due to relatively high yield, 2ng cfDNAs extracted using the Analytik kit was directly used for sequencing library construction. However, qualities of the sequencing libraries made from Analytik-derived cfDNA were extremely poor in three separate evaluation tests as determined by lack of featured library fragment band at ~300-310bp. Meanwhile, cfDNAs derived from Zymo kit generated consistent high quality sequencing library in three separate evaluation tests (Supplementary Figure 2).

### Urine cfDNA and sequencing library quality

The final cfDNA yield from 15ml urine samples ranged from undetectable (< 0.02ng/ul) to 1.6 ng/ul in 10ul elution buffer. Among 19 patients with both pre and post-treatment urine specimen, cfDNAs were detectable in 33 of the 38 samples. cfDNA yields from the remaining five samples were too low for measurement. For the 33 samples with total cfDNA > 0.25ng, sequencing libraries were prepared with final concentration of library DNAs between 0.878 and 3.490ng/ul. High sensitivity DNA chip showed multiple library fragments with peak size at ~300bp (Supplementary Figure 2). Whole genome sequencing generated approximately 7.6 million raw reads (ranging from 4.3 to 15.2) and 6.9 million mappable reads (ranging from 3.7 to 14.0). Corresponding mappable reads ranged from 77 to 93 percent of raw reads. The mean read count was ~134 per 60kb genomic window (Table 2).

**Table 2:** Statistics of whole genome sequencing

Sample ID	Raw Reads	Mappable Reads	Percent Mapped	Reads/60kb
1001U2	8,559,863	7,780,937	90.90	151
1002U1	5,834,360	5,359,747	91.87	104
1002U2	6,905,307	6,218,374	90.05	120
1003U1	8,466,006	6,717,216	79.34	130
1003U2	8,249,703	6,668,418	80.83	129
1004U1	5,695,192	5,239,912	92.01	101
1004U2	6,523,691	6,056,921	92.85	117
1005U1	8,028,779	7,146,997	89.02	138
1010U1	6,941,555	6,376,244	91.86	123
1010U2	8,066,311	7,392,163	91.64	143
1014U1	5,925,269	5,434,275	91.71	105
1014U2	5,709,509	5,264,081	92.20	102
1015U1	6,757,096	6,173,896	91.37	119
1015U2	5,082,468	4,680,443	92.09	91
1017U1	7,268,083	6,383,512	87.83	124
1017U2	8,751,507	7,962,944	90.99	154
1028U1	4,366,638	3,758,949	86.08	73
1028U2	6,505,105	5,927,164	91.12	115
1040U1	7,715,099	6,887,111	89.27	133
1040U2	8,481,528	7,888,688	93.01	153
1050U1	6,635,918	6,006,158	90.51	116
1043U1	7,398,308	6,791,284	91.80	131
1050U2	10,320,989	9,167,473	88.82	177
1059U1	15,250,498	14,061,397	92.20	272
1059U2	6,303,913	5,865,752	93.05	114
1060U1	10,515,071	9,691,459	92.17	188
1060U2	7,294,862	6,713,772	92.03	130
1080U1	9,525,919	8,793,277	92.31	170
1080U2	8,771,271	7,816,538	89.12	151
1084U1	10,930,339	10,145,924	92.82	196
1098U2	7,507,997	5,835,747	77.73	113
1104U1	5,670,174	5,231,642	92.27	101
1104U2	7,542,611	6,868,682	91.07	133
Mean	7,681,847	6,918,397	90.06	134

### Overall urine cfDNA genomic abnormalities

**Figure 1:**
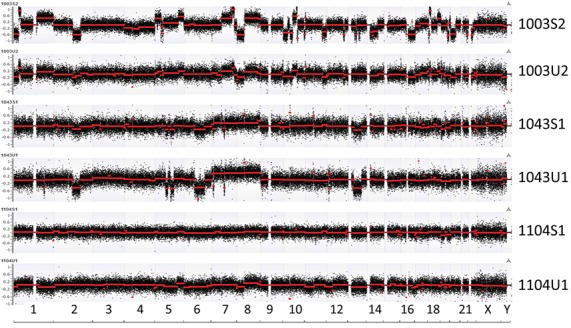
Overall view of genomic abnormalities in plasma (S) and urine (U) Log2 ratio-based segmentations across the human genome are shown with red line indicating averaged segments. Similar patterns of genomic abnormalities are seen in matched plasma and urine samples. “U1” represents the first urine sample findings for a particular patient identifier which is denoted by a four digit number. Similarly, “U2” represents the second serial urine specimen for the same patient; “S1” represents the first plasma specimen, while “S2” represents the second serial plasma sample.

To evaluate genomic abnormality, for each genomic bin, log2 ratio between read counts from urine cfDNA and lymphocyte-derived genomic DNA (gDNA) in the same patient was calculated. Fragmentation-based CNV analysis showed that cfDNA genomic abnormalities were detectable in all 19 patients tested. A greater number of genomic abnormalities were observed in the CRPC sub-cohort undergoing chemotherapy than in the HSPC cohort receiving ADT alone. Four of 10 CRPC patients (1003, 1004, 1014, 1017) and 2 of 9 HSPC patients (1050 and 1059) were observed to have specific genomic abnormalities. Of the 33 sequenced cfDNA specimens 14 patients had paired pre- and post-treatment cfDNA detectable abnormalities while five patients had either a pre- or post-treatment analysis. Among the 14 patients with paired samples, seven belonged to the HSPC sub-cohort and other seven to the CRPC sub-cohort. For these 14 patients, we performed unsupervised clustering analysis using log2 ratios in each genomic window and found that 11 pairs were clustered together (Supplementary Figure 3). Among these, some samples (such as patients 1050 and 1104) demonstrated significant CNV intensity differences between pre- and post-treatments. By comparing cfDNA-based CNVs from urine and plasma in matched patient samples, we observed consistent tumor-associated CNVs, although some differences of the log2 ratios in the two specimen types was observed (Figure 1).

### Genomic abnormalities at specific loci

**Figure 2:**
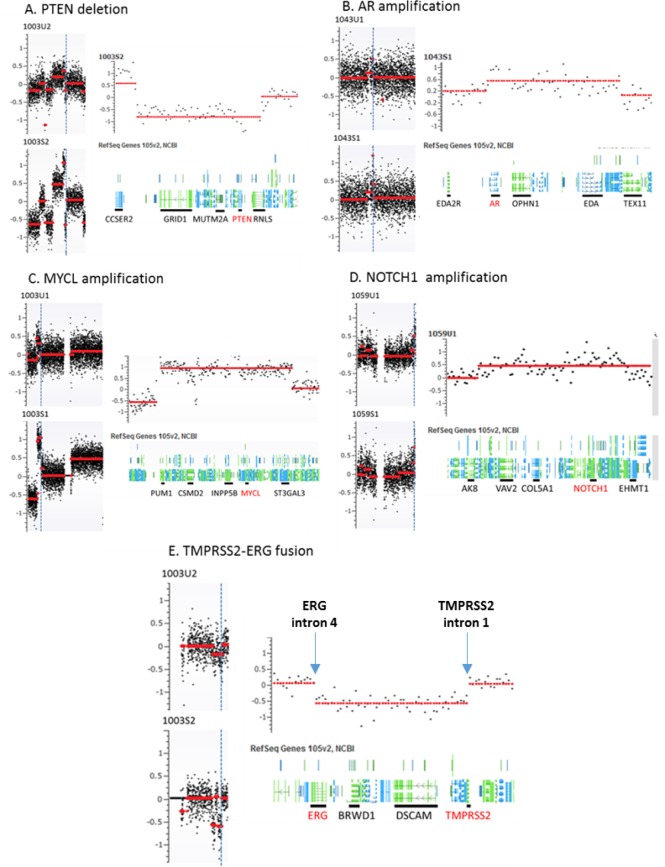
Representative genomic abnormalities detected at specific chromosomal loci in urine and matched plasma samples Panel **A.-E.** represent *PTEN* deletion at chr 10, *AR* amplification at chr X, *MYCL* amplification at chr 1 *NOTCH1* amplification at chr 9, and *TMPRSS2-ERG* fusion at chr 21. For each panel of A-E, left side shows chromosome level genomic changes and right side shows gene level genomic change at selected locus. 1003U2 and 1003S3 are urine and plasma cfDNAs from patient 1003, respectively. Vertical dot blue lines indicate the locations of these chromosomal aberrations.

To further define genomic abnormalities in urine, we performed detailed analysis at chromosomal regions with putative and frequent aberrations in prostate cancer. Among these, the androgen receptor (*AR*) genomic region is most frequently amplified in CRPC patients. To examine the amplification status, we zoomed to the genomic region containing the *AR* and observed *AR* locus amplification in five of ten CRPC cases (#1003, #1005, #1010, #1017, and #1043) but none in nine HSPC cases. Although the amplicon boundaries varied they all contained whole *AR* gene. Another common genomic aberration in prostate cancer is at *TMPRSS2* locus where frequent rearrangements create various fusion genes. We observed urine *TMPRSS2* genomic variations in four cases with CRPC (#1003, #1005, #1014 and #1017) and two cases with HSPC (#1040, and #1098). The breakpoints for two genomic losses occurred at the two gene (*ERG* and *TMPRSS2*) regions forming the *TMPRSS2-ERG* fusion gene. The third most common genomic abnormality observed in prostate cancer is *PTEN* deletion. We found the *PTEN* loss in four cases of our CRPC sub-cohort (#1002, #1005, #1043 and #1060) and one case of HSPC (#1080) in the urine cfDNAs. Additionally, we found *NOTCH1* locus amplification in one CRPC patients (#1014) and four HSPC patients (#1050, #1059, #1084 and #1098). Most of these abnormalities in urine cfDNAs were also observed and previously reported in the matched plasma cfDNAs [20] (Figure 2 and Table 3).

**Table 3:** Loss or gain of common prostate cancer-related genes

Gene	Deletion or Amplification	Urine	Plasma
PTEN	Deletion	1005U1,1043U1,1080U1,1060U2,1080U2,1002U1	1003S1,1005S1,1003S2,1060S1,1005S2,1043S2,1080S1
TMPRSS2	Deletion	1003U1,1003U2,1017U2,1005U1,1098U2,1040U1,1014U1	1003S1,1003S2,1043S1,1005S1,1005S2,1014S1
AR	Amplification	1003U2,1003U1,1005U1,1010U1,1017U1,1043U1	1003S2,1005S2,1010S1,1010S2,1028S2,1043S1,1060S1,1060S2
NOTCH1	Amplification	1059U1,1059U2,1098U2,1084U1,1050U1,1014U1	1059S1,1059S2
MYCL	Amplification	1003U1,1003U2,1104U1	1003S1,1003S2,1005S1,1059S2

**Figure 3:**
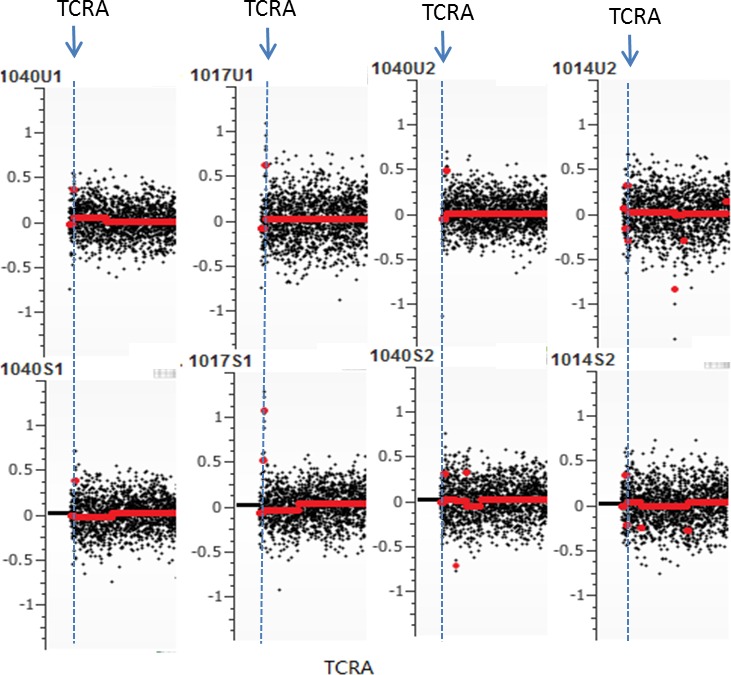
False positive (pseudo) amplification at *TCRA* locus Due to frequent rearrangements (deletions) of T cell-derived DNA at *TCRA* locus, use of lymphocyte DNA as normalization control may create pseudo copy number gain.

Other chromosomal regions were also frequently altered in the tested samples with most having at least one common deletion or amplification per chromosome. From the common regions, we further defined the minimally overlapped regions which were involved in amplifications at 8q24.3, 9q34.3, 11p15.5 and 14q11.2 and deletions at 4q35.2, 5q31.3, 7q36.3, 12q24.33, and 16p11.2 (Table 4). Among those, seven regions including 5q31.3, 7q36.3, 8q24.3, 9q34.3, 11p15.54, 14q11.2 and 16p11.2 have been reported to be associated with prostate cancer [21–26]. Meanwhile, gene mutations at these loci have also been reported in prostate cancer tissues [27–29]. In addition, frequent “amplification” at *TCRA* locus was observed in most urine samples. Because of extensive rearrangements (deletions) at *TCRA* locus during T cell development, lymphocyte-derived gDNA may harbor partial deletions at this locus. Consequently, using such gDNAs as controls to normalize cfDNA may generate false positive amplification at this locus (Figure 3).

**Table 4:** Co-deletion or co-amplification segment of minimal overlap region

Chr.	Start	Stop	Cytoband	Deletion or Amplification	Representative Genes	Sample ID (CRPC)	Sample ID (HSPC)
Chr4	189,361,876	191,048,841	4q35.2	Deletion		1060U1,1060U2,1010U2,1003U2, 1002U1,1002U2	1104U1,1104U2,1098U2,1080U1, 1080U2,1059U1,1059U2,1050U1
Chr5	140,501,206	140,700,782	5q31.3	Deletion	*NR3C1*	1060U1,1060U2, 1017U1,1005U1, 1001U2	1040U1,1040U2
Chr7	157,558,688	159,050,887	7q36.3	Deletion	*VIPR2*	1060U1, 1060U2	1104U1,1084U1, 1080U1,1080U2
Chr8	144,345,765	146,121,832	8q24.3	Amplification	*NDR1*	1014U1,1010U2, 1003U2	1104U1,1104U2,1098U2,1059U1,1059U2,1050U1
Chr9	139,266,197	140,278,759	9q34.3	Amplification	*NOTCH1, RXRA*	1002U1,1002U2, 1003U1,1010U2,1014U1	1050U1,1059U1,1059U2,1084U1,1098U2
Chr11	1	968,056	11p15.5	Amplification	*CD151, MUC6, MUC2, STIM1, CTSD, SLC22A18*	1060U1,1002U2,1003U1,1003U2,1014U1	1059U1,1059U2,1084U1,1050U1
Chr12	133,335,093	133,778,067	12q24.33	Deletion		1001U2,1017U2	1028U2,1040U2,1059U2,1080U1, 1080U2
Chr14	22,322,547	22,914,657	14q11.2	Amplification	*NDRG2, TCRA*	1043U1,1017U1,1017U2,1014U2, 1010U1,1010U2,1003U2	1084U1, 1040U1,1040U2
Chr16	33,889,263	33,988,937	16p11.2	Deletion	*TMS1*	1017U1,1017U2, 1005U1,1004U1,1004U2	1104U1, 1104U2, 1059U1, 1059U2, 1050U2,1028U1, 1015U2

### Urine genomic abnormality (UGA) score and its clinical utility

**Figure 4:**
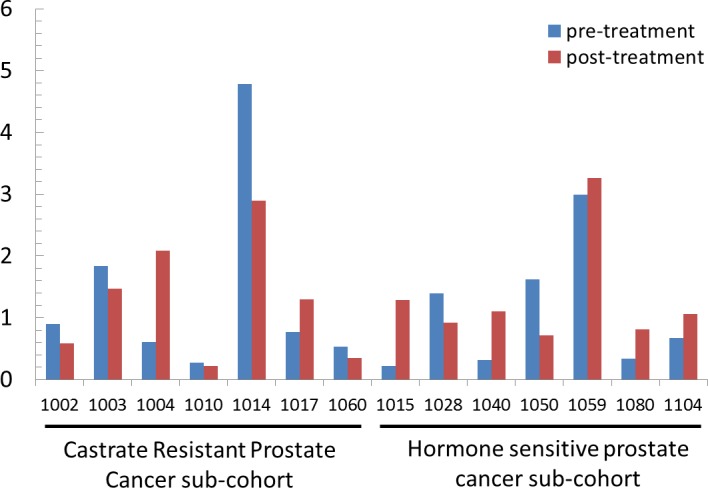
UGA score of 14 paired samples with pre- and post-stage specific therapies The UGA scores demonstrate inter- and intra-patient variations. Y-axis: Urine Genome Abnormality score.

**Figure 5:**
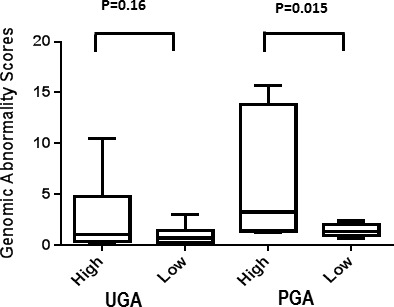
UGA and PGA score differences between high and low volume prostate cancer patients Average UGA score before treatment is lower in low volume patients than in high volume patients. Average PGA score before treatment is significantly lower in low volume patients than in high volume patients.

**Figure 6:**
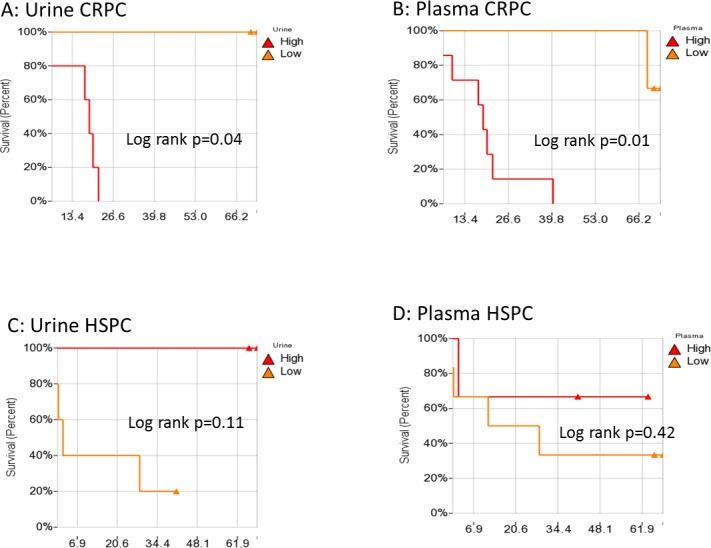
Kaplan-Meier analysis for the association of urine Teff **A.** and plasma Teff **B.** with overall survival in CRPC, and the association of urine Teff **C.** and plasma Teff **D.** with disease progression to CRPC. TEff: Treatment Efficacy index.

Previously, we calculated a plasma genomic abnormality (PGA) score based on multiple genomic abnormalities in plasma as a potential biomarker for association with treatment response and survival [20]. A similar UGA-based classifier was developed for which we modified the calculation of the previously reported PGA score algorithm. UGA score based inter and intra patient variations (for the 14 paired specimens) were observed (Figure 4) and UGA scores in the pre-treatment group were higher in patients with high volume disease than low volume disease although it did not reach statistical significance (*p* = 0.16) (Figure 5). To evaluate if a genomic abnormality change occurred after initiating treatments and was associated with clinical outcomes, the previously reported TEff (treatment effect) index an algorithmic score which compares the percent differences between pre and post-treatment genome abnormality scores was used [20]. Kaplan-Meier survival analysis showed that a higher TEff index was significantly associated with poor survival (*p* < 0.04) in CRPC cohort (Figure 6A and 6B). In the HSPC cohort, the UGA and/or the PGA based TEff index did not show an association with change in TEff index and progression to castrate resistance, although a higher TEff index was detected to have a statistically non-significant trend with longer progression time to castration resistance (Figure 6C).

### Treatment-associated genomic abnormalities

To examine treatment-associated genomic alterations, we generated log2 ratios between pre- and post-treatment specimens directly from scaled read counts at each genomic window and performed segmentation analysis for treatment-related genomic gain or loss. A total of 34 genomic loci with copy number changes were observed in the post treatment specimens. We then defined minimal overlap regions at each locus and identified commonly shared regions that covered nine genes (*ZNRF3, RNF43, LGR4, NCOR1, ZBTB16, MYC, FGFR1, KRAS and STK11*) (Supplementary Figure 4 and Table 5). For example, after treatment, the genomic region covering *LGR4* was amplified in two cases of HSPC (#1080 and #1104), and the genomic region covering *ZBTB16* was deleted in two cases of CRPC (#1014 and #1060). Copy number changes in the remaining seven gene regions were found in both advanced HSPC and CRPC urine specimens.

**Table 5:** Treatment-related genomic regions and genes

Chromosome	Location	Gene	Sample ID
Chr22	29,427,573- 29,453,476	ZNRF3	1003S2/S1(C*), 1050U2/U1(H*)
Chr17	56,431,037- 56,494,931	RNF43	1050U2/U1(H), 1014U2/U1(C)
Chr11	27,387,508- 27,494,338	LGR4	1104U2/U1(H), 1080U2/U1(H)
Chr17	15,933,864- 16,101,195	NCOR1	1060S2/S1(C), 1050U2/U1(H)
Chr11	113,933,133-114,126,702	ZBTB16	1060S2/S1(C), 1014U2/U1(C)
Chr8	128,748,449-128,753,674	MYC	1104U2/U1(H), 1060S2/S1(C)
Chr7	55,177,416- 55,279,262	FGFR1	1104U2/U1(H), 1060S2/S1(C), 1080S2/S1(H)
Chr12	25,358,180- 25,403,854	KRAS	1104U2/U1(H), 1060S2/S1(C), 1050U2/U1(H)
Chr19	1,205,798- 1,228,434	STK11	1104S2/S1(H), 1004U2/U1(C),1015U2/U1(H)

* C represents CRPC patients and H represents HSPC patients.

## DISCUSSION

cfDNA in blood has been extensively reported and proposed as biomarkers for cancer diagnosis, prognosis and treatment efficacy estimation. It is known that a small amount of cfDNA in blood passes into urine after renal filtration and tumor specific sequences are detectable in cfDNA isolated from urine [30, 31]. However a systematic determination of somatic genomic abnormalities in urine cfDNAs evaluated by high throughput sequencing technology in prostate cancer has not been performed [15, 19, 32]. Several challenges have limited this determination including a lack of precise knowledge on factors that may impact levels of urine cfDNA as cfDNA in urine is not as stable as in blood. In previous reports urine cfDNA profiling using PCR-based detection of candidate tumor-associated genes indicates that, an optimized and uniform method for cfDNA detection in urine that prevents degradation during extraction and storage should also include adequate volumes of specimens [33, 34]. Factors that may influence urine cfDNA detection include processing time of the urine samples after patient donation, the use of preservatives while processing, and the time of the urine samples in room temperature before storage in −80^°^C, and urine volumes.

We used a rigorous and uniform sample processing protocol for collecting 15ml of urine specimens and were able to detect cfDNA by Qubit instrument in most samples. Since the type of kit used for cfDNA extraction may impact yield and quality in this study we evaluated three commercial kits to identify association of extraction kit with cfDNA quality and yield. Although cfDNA yield using the Analytik kit was the highest, we were not able to apply the resultant cfDNA to generate high quality sequencing libraries. The Zymo kit generated relatively low yield but high quality sequencing libraries were consistently observed even at extremely low input of 0.25ng suggesting that selection of cfDNA extraction kit and thorough examination of cfDNA quality are important variables to consider for ensuring the subsequent success of sequencing library preparation and subsequent data analysis.

We were able to detect urine CNVs in all samples with adequate cfDNA quality and quantity, although the extent of detectable CNV per sample was stage-dependent with higher CNVs observed for CRPC patients than in HSPC stage. This finding is not surprising considering that mutations increase with progression in advanced prostate stages. CNV levels mirrored disease volume regardless of stage. We also observed lower level of detectable CNVs in urine specimens than in plasma (Figure 5), indicating a possible effect of renal filtration on urine cfDNA contents in concordance with previous reports in other tumor types [30, 31]. However, it does not appear to decrease the ability to detect specific somatic genomic changes. For example, shared specific genomic aberrations were observed in both plasma and urine cfDNAs at loci of *PTEN, TMPRSS2* and *AR* (Figure 2 and Supplementary Figure 5). These results suggest that both urine and plasma fractions can be used for developing liquid biopsy based biomarkers in advanced prostate cancer.

In this limited data set, urine cfDNA changes were also explored as predictive biomarkers by examining CNV changes after initiating treatment and were able to identify treatment-associated CNV changes at nine gene loci. Of interest, majority of gene loci identified have been reported to be aberrant in prostate cancer biology, such as copy number changes after treatment in *RNF43* and *ZNRF3 loci*. These two closely related single membrane spanning molecules have revealed receptor-like functionalities of a ligand-binding ectodomain. Combined with the intracellular architecture and activity of an E3 ligase, the two genes may be implicated in the modulation of *Wnt* signalling [35]. Post treatment copy number changes were also detectable in *LGR4* and *MYC* proto oncogene loci. *LGR4* has been reported to function in mammary gland development and mammary stem cells by activating *Sox2 via* the Wnt/β-catenin/Lef1 signaling pathway [36] and *MYC* proto-oncogene is frequently deregulated in prostate cancers, activating genetic programs that orchestrate biological processes to promote growth and proliferation [37].

Detection of cfDNA and the tumor-specific genomic aberrations in urine appears feasible and enhances the choices for developing liquid biopsy programs in advanced stage prostate cancer as predictive and prognostic classifiers. The approach adopted in our study performed with a limited number of samples for developing such classifiers is agnostic of specific gene/region changes and uses an algorithmic summation of the most common genetic abnormalities in urine. Since the mutational landscape of advanced prostate cancer is heterogeneous [38] this approach is likely to account for multiple genomic changes in tumor biology as a result of treatment effect. Due to small sample size, however, our findings are preliminary and need to be confirmed in larger cohorts of clinically annotated specimens. In addition, current whole genome-based sequencing technology is not sensitive to detect low level genomic abnormality. Its low sequencing depth makes detection of genomic rearrangement difficult. Regardless, with rapid advances in high throughput sequencing technology, sensitive detection of low level tumor-associated cfDNAs in body fluids will become feasible [39]. Urine cfDNA-based genomic abnormality tests may have the potential to provide a measurable classifier that can be used to assess treatment response and clinical outcomes in advanced prostate cancer patients.

## MATERIALS AND METHODS

### Patient methods

Urine specimens were obtained from advanced prostate cancer patients in metastatic hormone sensitive and metastatic castrate resistant stages. Patients were enrolled in a prospectively collected, institutional review board (IRB) approved study at a tertiary hospital while undergoing stage-specific standard of care treatments. Informed consent was obtained from all patients enrolled in the registry. The primary purpose of the registry is for developing blood and urine-based classifiers of disease and treatment outcomes in this patient population while patients receive standard of care treatments. Twenty cases (ten hormone sensitive and ten castrate resistant stage patients) were selected for this study with each patient having two serial urine samples. Each patient provided the first of the two urine specimens before initiating stage specific treatment and a second specimen after starting treatments. All cases selected for this study had matched plasma cfDNA sequencing performed previously [20]. All urine specimens were collected at the same time as the plasma collections. Initial processing of all urine specimens was performed uniformly within 45 minutes of receiving the sample from the patient. An initial centrifugation at 600g for 10 minutes was followed by storage of the urine and pellet in −80^°^C. No urine specimen underwent any freeze-thaw cycles other than at the time of extraction of cfDNA. Peripheral blood mononuclear cell-derived germline DNA (gDNA) was collected at the same time as the plasma and urine specimens. Clinical outcomes of patients undergoing this prospective specimen banking was performed retrospectively as previously described [20].

### Isolation of cell free DNA (cfDNA)

To determine the best urine cfDNA extraction kit, we tested three different commercial products using a single urine sample. The kits included Extract-all Urine DNA kit (Zymo research corp., CA, USA), Urine DNA isolation kit (Norgen Biotek Corp., Ontario, Canada), and PME free-circulating DNA Extraction kit (Analytik Jena Innuscreen GmbH, Berlin, Germany). After thawing the urine sample, it was placed on ice immediately and then centrifuging of 15 ml urine was performed at 3000rpm for 15 minutes. The supernatant was used for DNA extraction according to each manufacturers' protocol. cfDNA was eluted in 30ul elution buffer and concentration was measured using Qubit Fluorometer (Life Technology, Carlsbad, CA).

### DNA extraction and sequencing library preparation

After an initial evaluation of the yield and quality of cfDNA from the three commercial kits, the Zymo research urine DNA Kit (Zymo Research, Irvine, CA) was selected to extract cfDNAs from 15 ml according to the manufacturer's instructions. The extracted DNA was eluted in 10ul water. 1ul DNA eluent was quantified using Qubit. The remaining was stored at −80^°^C until preparation of sequencing libraries. For each patient germline DNA (gDNA) was also extracted and quantified. Sequencing DNA libraries were prepared for the urine cfDNA using a ThruPLEX DNA-Seq Kit (Rubicon Genomics, Inc. Ann Arbor, MI). 24 indexed libraries were pooled for single-read sequencing on a HiSeq2000 Sequencing System (Illumina, San Diego, CA).

### Copy number variation (CNV) calculation

Raw sequencing data (fastq files) were first mapped to the human genome (hg19) (DNASTAR, Madison, WI). Read counts from the mapped sequence files were then binned into 60kb windows (total 51672 genomic bins) and adjusted to the global mean count for each sample. The read count ratio in each genomic bin was calculated by dividing cfDNA with peripheral blood mononuclear cell germline DNA (gDNA) in the same patient [20]. The resulting ratios were further transformed with log2 and corrected for GC content [40]. The fully normalized log2 ratios in genomic bins were subjected to segmentation using the copy number analysis method (CNAM) algorithm (Golden Helix, Bozeman, MT).

### Urine genome abnormality (UGA) score algorithm calculation and comparison with plasma genome abnormality score (PGA)

To quantify genomic abnormality we modified the previously reported methodology for calculating global genomic abnormalities in plasma (plasma genome abnormality, PGA) score [20]. This was performed by summing the most significant log2 ratios in top 95-99% genomic bins. For the current study, we modified the genome abnormality calculation by summing log2 ratios of ten most significant genomic segments. To generate the UGA score, we first mapped raw sequencing data (fastq files) to the human genome (hg19) (DNASTAR, Madison, WI). We then binned read counts from the mapped sequence files into 60kb windows (total 51607 genomic bins) and rescaled to the global mean count for each sample. To account for constitutional CNVs, we divided read count in each bin from urine cfDNA by the one in the same bin from gDNA to generate a normalized read count ratio. The resulting ratio was transformed by log2 transformation and GC content correction [40]. Finally, we performed segmentation analysis using the copy number analysis method (CNAM) algorithm (Golden Helix, Bozeman, MT). Due to highly repeated sequences in centromeres, we removed genomic regions containing centromeres and their surrounding +/−1Mb regions. We also excluded small genomic segments containing ≤ 4 bin windows (4×60kb). From the remaining segments, we summed the top ten most significant segment values (using absolute numbers) and defined the summarized number as Urine Genomic abnormality (UGA) score. For consistency and comparability the previously reported PGA score [20] was also re-analyzed in the same manner as the UGA. A higher score being indicative of a greater tumor DNA fraction in the cfDNA. To quantify a treatment response index in each patient, we defined the TEff (Treatment Efficacy) index as the log2 ratio of UGA (or PGA) scores between the pre- and post-treatments: TEff index = log2 (prePGA/postPGA) x100.

### Statistical analysis

For defining hormone sensitive and castrate resistant stage in this hospital based registry a uniform definition was used as reported previously [20, 41]. Briefly, for the CRPC cohort, overall survival was recorded from the date of first plasma collection after ADT failure to death or last follow-up. For the HSPC cohort, disease progression was recorded from the date of first plasma collection at initiation of ADT to disease progression or last follow-up. To evaluate association of the UGA score with overall survival in the CRPC sub-cohort, time from developing castrate resistance to death was considered and Kaplan-Meier analysis was performed for the UGA score and TEff index associations with overall survival (prognostic classifier). For the HSPC cohort time from initiating androgen deprivation therapy (ADT) for hormone sensitive stage to development of castrate resistance was obtained (predictive classifier). We dichotomized each sub-cohort into two risk groups using median UGA score or TEff index as a cut-off. A P-value of ≤ 0.05 was considered statistically significant for all statistical analyses.

## SUPPLEMENTARY MATERIAL FIGURES


