# Supplementary material for: Copy number variations in urine cell free DNA as biomarkers in advanced prostate cancer
Source: Oncotarget. 2016 Apr 26;7(24):35818–31. doi: 10.18632/oncotarget.9027 (PMC5094965; doi:10.18632/oncotarget.9027)
Supplement: Supplementary file 1 [file oncotarget-07-35818-s001.pdf]

Copy number variations in urine cell free DNA as biomarkers in advanced prostate cancer

Supplementary Material

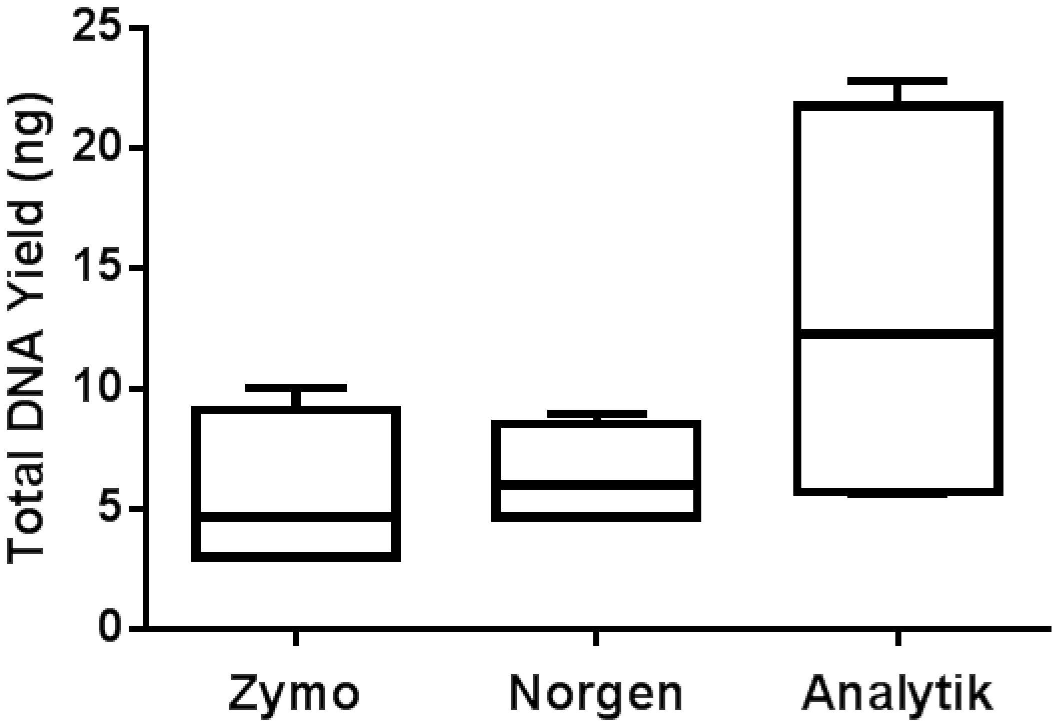

Supplementary Figure 1. Final cfDNA yields from 15 ml normal adult male urine by three different kits (Zymo, Norgen, and Analytikjena).

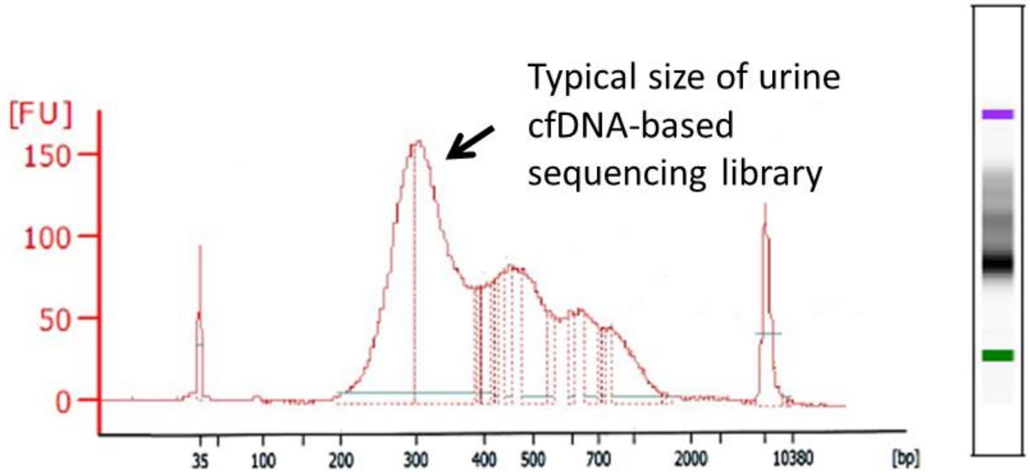

Supplementary Figure 2. Representative electropherogram of urine cfDNA sequencing library. Fragment sizes after adding sequencing adaptors range from 250 to 1500bp with peak at ~ 300bp.

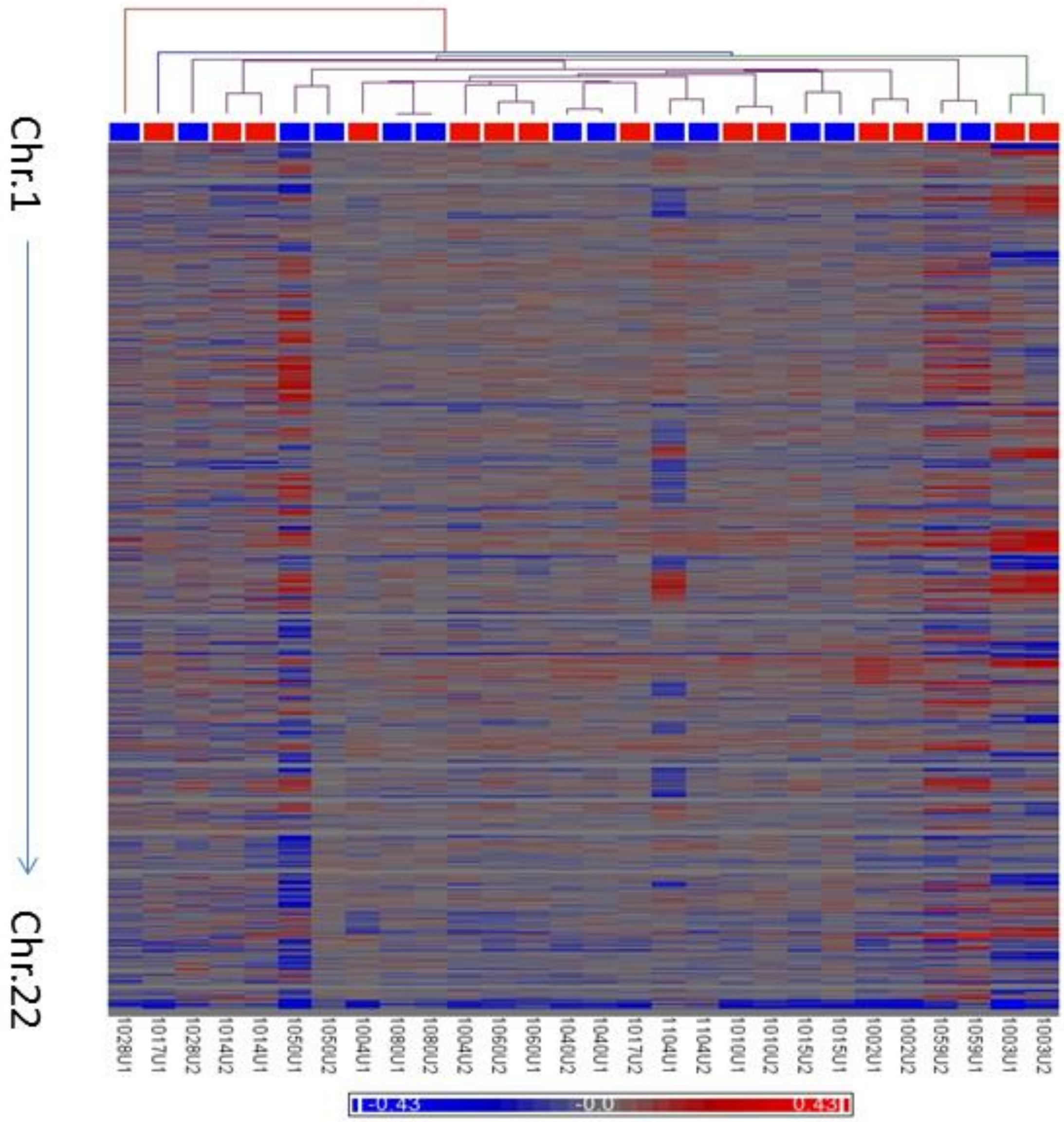

Supplementary Figure 3. Log2 ratio-based clustering analysis in 14 urine pairs before and after stage-specific therapy. U1 and U2 are urine collection time 1 and 2, respectively.

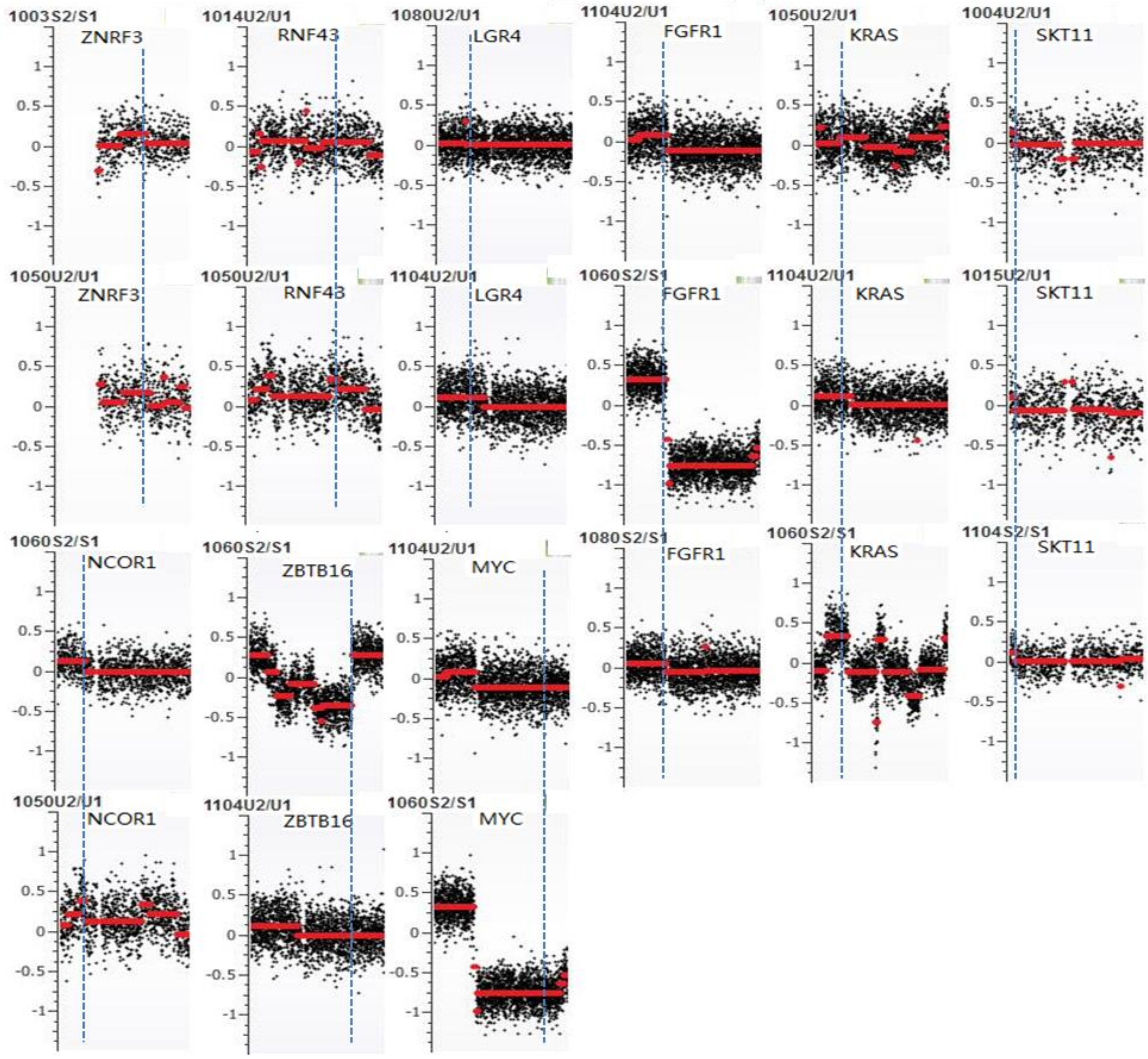

Supplementary Figure 4. Treatment-related genomic regions and genes. Chromosomes harboring the common genomic regions are shown. Horizontal red lines in each chromosome are trend line (segment) of copy number changes. Vertical blue lines indicate the location of key genes.

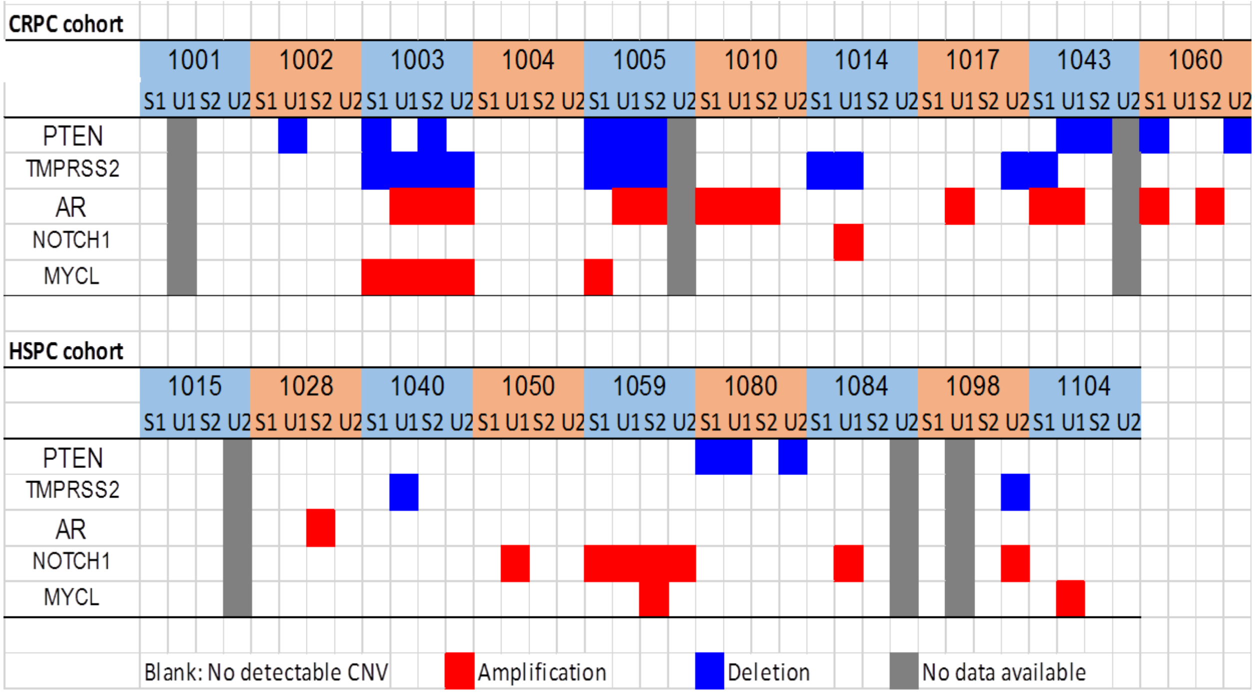

Supplementary Figure 5. Selected CNV based genomic abnormalities per visit detected in urine and plasma for 19 patients
